# Exploring the unusual: a testosterone-secreting ovarian tumor

**DOI:** 10.4322/acr.2024.478

**Published:** 2024-02-26

**Authors:** Harpreet Kaur, Neha Singh, Sushma Bharti, Gurwinder Kaur

**Affiliations:** 1 All India Institute of Medical Sciences (AIIMS), Department of Obstetrics and Gynecology, Bilaspur, Himachal Pradesh, India; All India Institute of Medical Sciences (AIIMS), Department of Pathology, Bilaspur, Himachal Pradesh, India

**Keywords:** Ovarian neoplasm, Sex Cord-Gonadal Stromal Tumours, Hirsutism, Postmenopause.

## Abstract

Ovarian steroid cell tumors are rare, representing less than 0.1% of all ovarian neoplasms. Among the myriad causes of hirsutism, ovarian tumors account for 1% of the reported cases. We present the case of a 49-year-old parous postmenopausal woman who sought medical attention for hirsutism for 2 years. This case illustrates the unusual and interesting connection between rare ovarian pathology and the clinical manifestation of hirsutism in a postmenopausal patient. Her ultrasonography and MRI showed a right adnexal mass of solid-cystic consistency with thin septations. Her laboratory workup revealed high levels of total testosterone of 256 ng/ml (8.4-48.1ng/ml) and free testosterone of 7.36 pg/ml (0.2-4.1 pg/ml), while DHEAS - 234 µg/dl (35.4-256 µg/dl) and CA125 - 15.8U/L (0.0-35 U/L) were in the normal range. She underwent exploratory laparotomy with a total abdominal hysterectomy and oophorectomy. Histopathological examination and immunohistochemistry conclusively established the presence of a steroid cell tumor, specifically classified as "Not Otherwise Specified"(NOS), in the right ovary.

## INTRODUCTION

Sex cord-stromal tumors account for approximately 5-8% of all ovarian neoplasms.^1^ In this subset of tumors, the ovarian steroid cell tumor is a rare functioning sex cord-stromal tumor. They comprise <0.1% of all ovarian tumors.^2^ In earlier days, these were called lipoid cell tumors of the ovary. In adult women, testosterone is produced by the ovaries and adrenal glands and through indirect conversion from androgen precursors in peripheral tissue, including muscle, fat, and skin. Elevated testosterone can be seen in several disorders, such as polycystic ovarian tumors, exogenous testosterone treatment, and ovarian and adrenal gland tumors. Hyperandrogenism of adrenal origin is frequently associated with raised serum cortisol and dehydroepiandrosterone sulfate levels 

A subtype of "Sex cord-stromal tumor NOS’’ accounts for one-half of all steroid cell tumors. Approximately one-third of steroid cell tumors in adults have been reported to be malignant. In the context of hirsutism, which is assessed clinically by the Ferriman Gallwey score,^3^ it's quite remarkable that ovarian tumors are responsible for 1% of all reported cases. This emphasizes the rarity of hirsutism linked to ovarian tumors and highlights the unique nature of this clinical presentation.^4^

## CASE REPORT

**Figure 1:**
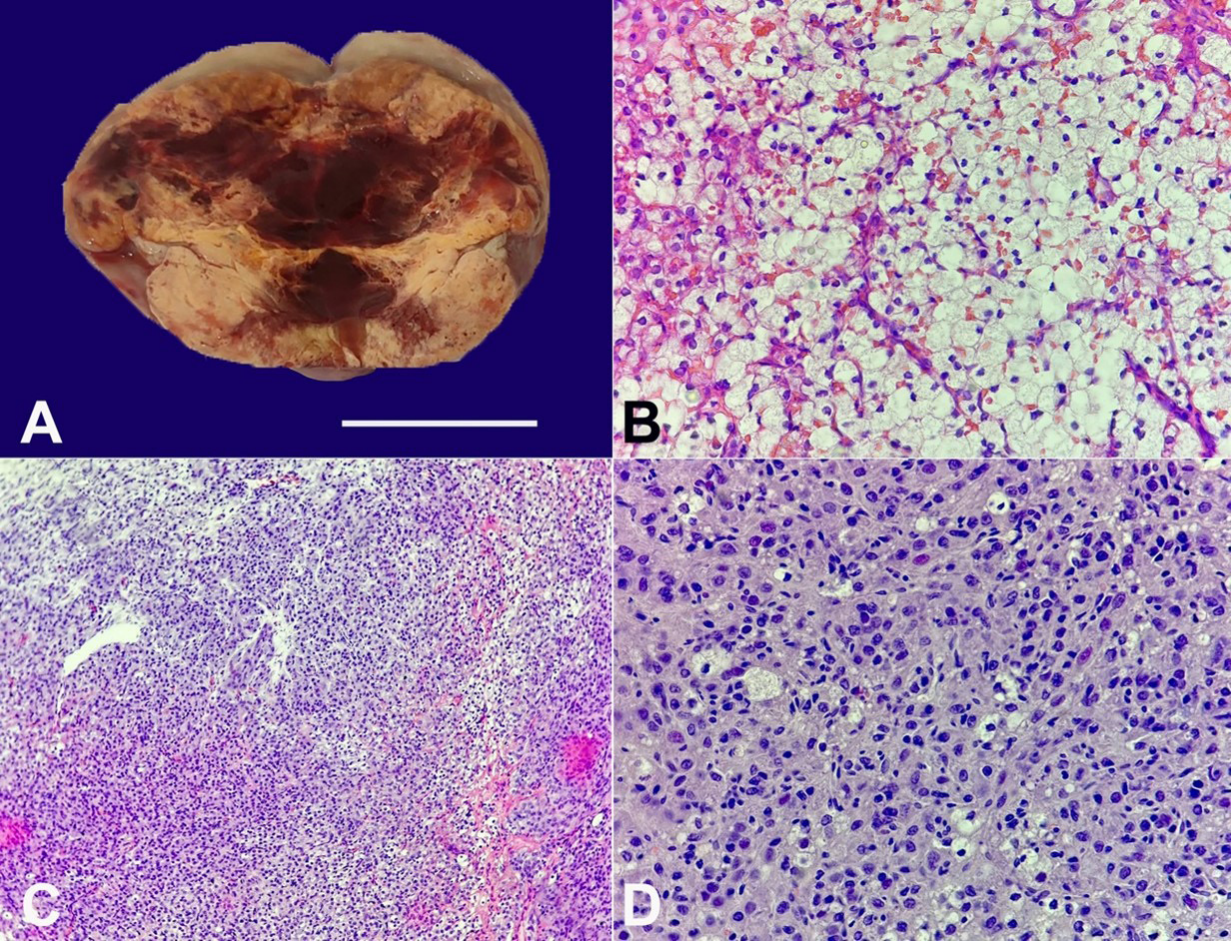
**A** - Gross view of the cut surface of the yellow to orange ovary with areas of hemorrhage; **B -** Frozen section: showing the sheets of tumor cells having round to oval monomorphic nuclei and abundant eosinophilic to vacuolated cytoplasm (H&E, 40x); **C -** Paraffin section: showing sheets and clusters of tumor cells with scant intervening stroma (H&E, 10x); **D -** Paraffin section: Cells are polyhedral having central nuclei, prominent nucleoli and abundant eosinophilic to clear multivacuolated cytoplasm (H&E, 40x).

A 49-year-old parous postmenopausal lady presented with complaints of increasing hair growth over the chin and neck region after 2 years of menopause. On examination, she had a BMI of 27 Kg/m^2^, a Ferriman-Gallwey score of 20, and on vaginal examination, was found to have a right adnexal mass 5 x 4 cm. She was already on treatment for hypertension, diabetes, and hypothyroidism. Her USG and MRI showed a uterus with mild myometrial heterogenous echotexture and a right solid-cystic adnexal lesion with thin septations of 5.4 x 4.6 x 4.5cm. Laboratory investigations revealed CA125 - 15.8 U/L (0.0-35 U/L), Total testosterone - 256 ng/ml (8.4-48.1 ng/ml), free testosterone - 7.36 pg/ml (0.2-4.1 pg/ml), DHEAS - 234 µg/dl (35.4-256 µg/dl), LDH -172 U/L (135-214 U/L), AFP-1.67 ng/ml (<7ng/ml), CEA-0.96 ng/ml (0.0-4.7 ng/ml), inhibin B - 25.22 pg/ml (5-33 pg/ml) and serum cortisol-13.50 (within normal limit). The breast ultrasound was normal. Given the clinical diagnosis of a hormone-secreting tumor, she was planned for exploratory laparotomy and total abdominal hysterectomy with right oophorectomy. Intraoperatively, the left ovary and bilateral tubes were absent as the patient had a previous history of surgery for family planning. Minimal ascitic fluid was found, negative for malignant cells on cytology. The right ovary was grossly enlarged and cystic, measuring 5x6 cm with an intact capsule. (Figure 1A)

The frozen section of the oophorectomy showed a benign sex cord-stromal tumor (Figures 1B and 1C). The histopathological examination revealed the presence of a steroid cell tumor in the right ovary, specifically classified as "Not Otherwise Specified" (NOS). Notably, there were no indications of nuclear atypia or mitotic activity, suggesting a relatively benign nature of the tumor (Figure 1D). 

**Figure 2:**
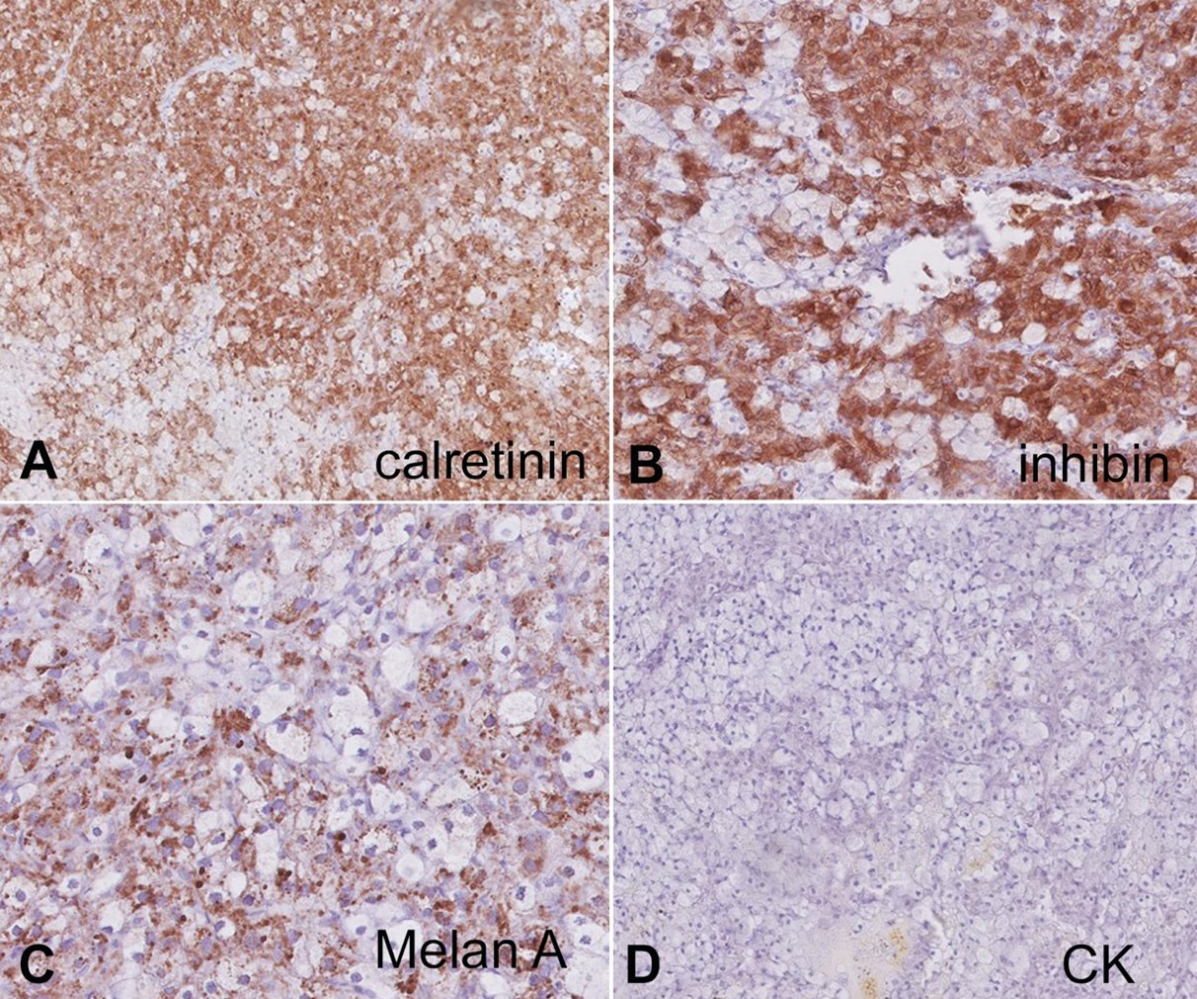
Photomicrographs of the tumor. **A**, **B**, and **C -** Immunohistochemistry diffuse nuclear and cytoplasmic staining for calretinin, Diffuse granular cytoplasmic staining for Inhibin, focal Granular cytoplasmic staining for Melan A (40X). **D** - Immunohistochemistry is negative for Cytokeratin (40x).

The examination of the uterus and endometrium revealed unremarkable findings, with evidence of adenomyosis. Immunohistochemistry was performed, and the results showed immunoreactivity for inhibin, calretinin, and Melan-A. (Figure 2A, 2B, 2C) Additionally, there was patchy immunoreactivity for vimentin. Notably, there was no reactivity observed for Pan CK (pan-cytokeratin) (Figure 2D) and PAX8, which collectively supported the diagnosis of an ovarian steroid cell tumor classified as "Not Otherwise Specified" (NOS). 

These immunohistochemical findings further strengthened the characterization of the tumor type. The post-operative course was unremarkable. The patient is doing well three months post-surgery, with serum total testosterone levels falling to 15.60 ng/dl. 

In the current case, serum testosterone level returned to normal after 6 weeks of surgery.

Ovarian steroid cell tumors are rare, which makes early detection challenging. However, this case highlights the critical importance of vigilance in assessing unusual hormonal symptoms, such as hirsutism. Clinicians should maintain a high index of suspicion for such tumors in cases of atypical hormonal presentations. 

## DISCUSSION

Ovarian Sex cord-stromal tumors are rare and usually occur in the first two to three decades of life.^5-7^ However, exceptions such as granulosa cell tumors typically emerge between 50-55 years of age.^5^ These tumors represent approximately 5-8% of all primary ovarian tumors.^8^ They are classified within a broader category of ovarian tumors known as sex cord-stromal tumors, which constitute a heterogeneous group of both benign and malignant neoplasms. These tumors originate from distinct cell types that develop from stromal cells or primitive sex cord cells, contributing to their diversity.^9,10^ The stromal cells encompass theca cells, fibroblasts, and Leydig cells, while the gonadal primitive sex cords comprise granulosa cells and Sertoli cells.^11^ Sex cord-stromal tumors include several subtypes with unique characteristics and behaviors. The World Health Organization (WHO) updated the classification of sex cord-stromal tumors in a significant revision in 2014.^12^ Sertoli-Leydig Cell Tumors arise from primitive sex cord cells and can produce androgens, resulting in masculinization or virilization in affected patients. They are rare but can occur in a wide age range.^10^ Thecoma-Fibroma Group are typically benign tumors. They often present with symptoms related to their size, such as abdominal discomfort.^13^ Steroid cell tumors typically manifest around the age of 40 on average.^11^ These tumors are characterized by their origin in the ovarian stromal tissue and their ability to produce hormones, particularly steroids. They can be further classified into two main subtypes: stromal luteoma and Leydig cell tumor, with the latter being more common.^14^ Most of these tumors are androgenic, with approximately 50% of patients displaying virilizing symptoms. On rare occasions, these tumors may also be linked to estrogenic manifestations, and there have been a few reported cases associated with hypercortisolism and pregestational alteration.^11,12,15^

Progressive hirsutism presenting in postmenopausal years requires a detailed evaluation. In the current case, serum testosterone was 256 ng/ml, and DHEAS level and serum cortisol level were normal, which helped us rule out the possibility of Cushing syndrome and an androgen-producing adrenal tumor.^16^ In the evaluation of hyperandrogenism, the initial steps of the diagnosis involved the assessment of serum testosterone and DHEA-S levels. These tests are essential for distinguishing between adrenal and ovarian sources of pathological androgen production, particularly in cases of hirsutism. Serum testosterone level >200ng/dl is an important diagnostic threshold level for the discrimination of neoplastic sources from other nonneoplastic causes of hirsutism. Therefore, hormonal assessments help in guiding the diagnosis and further treatment. Most virilizing tumors are ovarian in origin and include Sertoli-Leydig cell tumors, hilus cell tumors, steroid cell tumors, and infrequently granulosa theca cell tumors. In rare instances, non-functional ovarian neoplasms like epithelial cystadenomas or cystadenocarcinomas can also lead to androgen excess by stimulating steroid production in the surrounding nonneoplastic ovarian stromal tissue. 

The ovarian steroid cell tumor was first described by Scully, who reported 63 cases ranging from 2 to 80 years of age.^17^ Steroid cell tumors have been classified into three subtype-NOS, Leydig cell tumor, and stromal luteoma.^18^ Ovarian steroid cell tumor-NOS is typically composed of round to polygonal cells with well-defined cell membranes containing abundant eosinophilic or vacuolated cytoplasm. These cells can form solid sheets, nests, or cords within the ovarian tissue. Their nuclei often appear round to oval and may contain prominent nucleoli.^14^ The immunohistochemistry is a valuable technique in diagnosing and confirming the diagnosis. Commonly used markers in ovarian steroid cell tumors include inhibin, calretinin, and Melan A.^19^ Inhibin, produced by granulosa cells and steroidogenic cells, is frequently expressed in ovarian steroid cell tumors, offering a crucial diagnostic clue. Calretinin, another useful marker, often shows positive staining in these tumors.^20^

In a series of cases from Massachusetts General Hospital, 94% of the tumors were found to be unilateral, and 28.6% were malignant.^18^ Pathologically moderate to marked nuclear pleomorphism, necrosis, hemorrhage, vascular and capsular infiltration classify the tumor as malignant. Steroid cell tumor NOS Malignant NOS steroid cell tumors should be managed with surgical removal followed by a combination of chemotherapy and radiotherapy.^21^ The management of a patient with an ovarian steroid cell tumor involves a multifaceted approach depending upon the specific tumor characteristics. Most of these tumors are diagnosed at an early stage due to earlier presentation of clinical signs and symptoms, and they usually do not recur or metastasize. So, the therapeutic value of chemotherapy and radiotherapy is controversial.^22^ The primary treatment for ovarian steroid cell tumors is surgical removal. It mainly involves a unilateral salpingo-oophorectomy. Surgical removal may be sufficient to provide a complete cure in cases where the tumor is benign. In the index case, total abdominal hysterectomy with oophorectomy was done as the patient was postmenopausal and had no desire for future childbearing.

However, postoperative follow-up assessment, which includes the measurement of sex hormone levels, is necessary.^22^ In malignant or aggressive variants, a more extensive procedure such as a total abdominal hysterectomy and bilateral salpingo-oophorectomy may be necessary to ensure complete tumor removal and prevent a recurrence. Unfortunately, there isn't enough data to recommend a specific method.^22^ During surgery, it's essential to assess the extent of the disease and check for potential spread. The role of lymph node removal is controversial, but in suspected malignancy, lymph node sampling may be required to evaluate for metastasis.^5,10^ The decision to administer chemotherapy is typically based on the tumor’s characteristics and the disease extension. The 5-day bleomycin, etoposide, and cisplatin (BEP) regimen is the most commonly employed first-line chemotherapy combination for steroid cell tumors. Nonetheless, the literature offers limited reports regarding chemotherapy for steroid cell tumors, and there is no conclusive evidence of its effectiveness.^14^ In cases of hormonally active tumors, such as those producing excess androgens, hormonal therapy can be given. These may include the administration of medications to suppress the symptoms of androgen excess, like hirsutism or virilization.^4^ The choice of medication depends on the patient's age, fertility considerations, and the specific hormonal profile. After initial treatment, long-term follow-up is crucial. Regular clinical and imaging evaluations are essential to monitor for any recurrence or late complications. Additionally, hormonal assessments are vital to ensure that androgen levels are within the normal range.^6^ Hyperandrogenism in postmenopausal females should always be investigated, and malignancy needs to be ruled out. Rapid progression of symptoms and abdominal signs might point towards a suspicious entity, which can be corroborated by the USG and MRI imaging. Obtaining a frozen section is considered an important clinical practice, as it determines the optimal scope of surgical intervention. In addition, histopathology and immunohistochemistry play a crucial role in diagnosing complex cases, enhancing diagnostic accuracy, and resulting in better treatment planning. The rationale behind using histomorphology and immunohistochemistry is that while the morphological appearance can raise suspicion of a steroid cell tumor, immunohistochemistry helps confirm the diagnosis with higher specificity; in particular, the positive staining of inhibin and calretinin in the tumor cells is considered a hallmark of ovarian steroid cell tumors.^19,20^

 The prognosis for ovarian steroid cell tumors can vary widely and is influenced by several factors, including the patient's age, tumor size, grade, and stage.^7^ These tumors are often benign and have an excellent prognosis.^10^ Good prognosis for ovarian steroid cell tumor NOS includes young age, benign or low-grade histology, no evidence of metastatic disease, and small size. 

The accurate diagnosis of ovarian steroid cell tumors depends on histopathology and immunohistochemistry.^19,20^ This case highlights how these diagnostic tools provide essential information to differentiate this rare tumor from more common gynecological neoplasms. Combining specific morphology and immunohistochemical markers, such as inhibin and calretinin, allows for a precise diagnosis. 

Steroid cell tumor management varies depending upon the benign or malignant nature. Benign tumors can be cured with surgery removal only, whereas malignant tumors require adjuvant chemotherapy.^22^ Therefore, a multidisciplinary team effort comprising gynecologists, pathologists, radiologists, and endocrinologists is crucial to managing such cases. 

## CONCLUSION

In summary, this case highlights the importance of considering testosterone-secreting ovarian tumors in postmenopausal women presenting with hirsutism. Timely diagnosis, surgical intervention, and appropriate postoperative monitoring can lead to symptom resolution and a favorable outcome in these rare cases.
